# Autoimmune gastritis diagnosed due to recurrent gastric neuroendocrine tumor: a case report

**DOI:** 10.3389/fmed.2024.1519819

**Published:** 2025-01-03

**Authors:** Qunying Yang, XiangHong Jin, Xiangyin Lv, JianWen Hu

**Affiliations:** Department of Gastroenterology, Dongyang People's Hospital, Dongyang, China

**Keywords:** autoimmune gastritis, atrophic gastritis, gastric neuroendocrine tumors, endoscopic submucosal dissection, gastrin

## Abstract

As digestive endoscopy becomes more prevalent, an increasing number of autoimmune gastritis (AIG) cases have been diagnosed, which has contributed to a growing body of research on AIG. We report the case of a patient with AIG who was diagnosed due to receiving endoscopic surgery after discovering a gastric neuroendocrine tumor (GNET) during gastroscopy twice within 3 years. The patient was admitted to our hospital for endoscopic submucosal dissection (ESD) due to GNET recurrence discovered during gastroscopy. The patient had previously undergone ESD due to a GNET discovered during gastroscopy 3 years ago. Recent repeat gastroscopy revealed severe mucosal atrophy in the gastric body and fundus, an ulcer in the gastric antral, and two mucosal bulges in the gastric body. Pathology indicated Grade 2 (G2)-GNET, and ESD was performed again. The patient also had iron deficiency anemia and thyroid dysfunction, elevated gastrin, and decreased pepsinogen I (PG I) and PG I/II. Hence, AIG was diagnosed. Recurrent GNET cases, especially those with concurrent anemia and abnormal thyroid function, may experience AIG. In addition to symptomatic treatment, the clinician must evaluate the patient's overall condition.

## 1 Introduction

Despite the prevalence of gastric cancer screening and the improved eradication rate for *Helicobacter pylori*, there has been a rise in the cases of autoimmune gastritis (AIG) diagnosed, which is now not an uncommon disease. However, research suggests that the diagnosis of AIG is severely delayed and fraught with challenges (1–3). AIG is a chronic immune-mediated disease of the gastric mucosa. It can cause progressive atrophy and sequential functional loss of the gastric oxyntic glands, resulting achlorhydria and achlorhydria-induced hypergastrinemia (4). Type 1 gastric neuroendocrine tumors (GNETs) are tumors derived from enterochromaffin-like (ECLs) cells. They are thought to be associated with hypergastrinemia, as gastrin can stimulate the proliferation, hyperplasia and atypical hyperplasia of ECL cells, thereby driving the formation of GNETs (5, 6). Gastroscopy and endoscopic surgery are crucial methods for screening and treat in GNET in patients with AIG (7). Here, we report the case of a patient who was diagnosed with AIG due to GNET discovered during two gastroscopy examinations within 3 years. We aim to remind clinicians to take note of the link between AIG and GNET, thereby improving the diagnosis rates of AIG rather than merely providing symptomatic treatment.

## 2 Case description

A 53-year-old female was admitted to the hospital 11 days after discovering a gastric tumor. Eleven days before admission, the patient underwent a gastroscopy examination, which revealed chronic atrophic gastritis, an ulcer at the gastric antrum, and two mucosal bulges in the gastric body. A biopsy of the lesion in the gastric body indicated G2-GNET. The patient underwent endoscopic submucosal dissection (ESD) 3 years ago due to a GNET discovered during gastroscopy. The patient also had hypothyroidism and received thyroxine supplements, but the medication was discontinued in the past 2 months. The patient's father had a history of hypertension, and her mother had a history of hypertension and type 2 diabetes.

Vital signs at admission: body temperature 36.5°C; blood pressure 118/67 mmHg; heart rate 80 beats/min; respiratory rate: 18 breaths/min. The patient also had an anemic appearance and pale skin and mucosa. The patient showed normal white blood cells and platelets, reduced hemoglobin (93 g/L), reduced serum iron (3.8 μmol/L), reduced vitamin B12 (114 pmol/L), and normal folic acid and ferritin. She also showed elevated gastrin (328 μg/L), reduced pepsinogen (PG) I (10.2 μg/L), normal PG II (10.9 μg/L), and reduced PG I/II (0.94). All tumor indicators were normal, thyroxine level was normal, thyrotropin was slightly elevated (5.91 μIU/mL), and fecal occult blood was negative. On March 2, 2021, the patient received gastric ESD due to a GNET discovered during gastroscopy in another hospital. Postoperative pathology and immunohistochemistry indicated Grade 1 (G1)-GNET.

**Figure 1:**
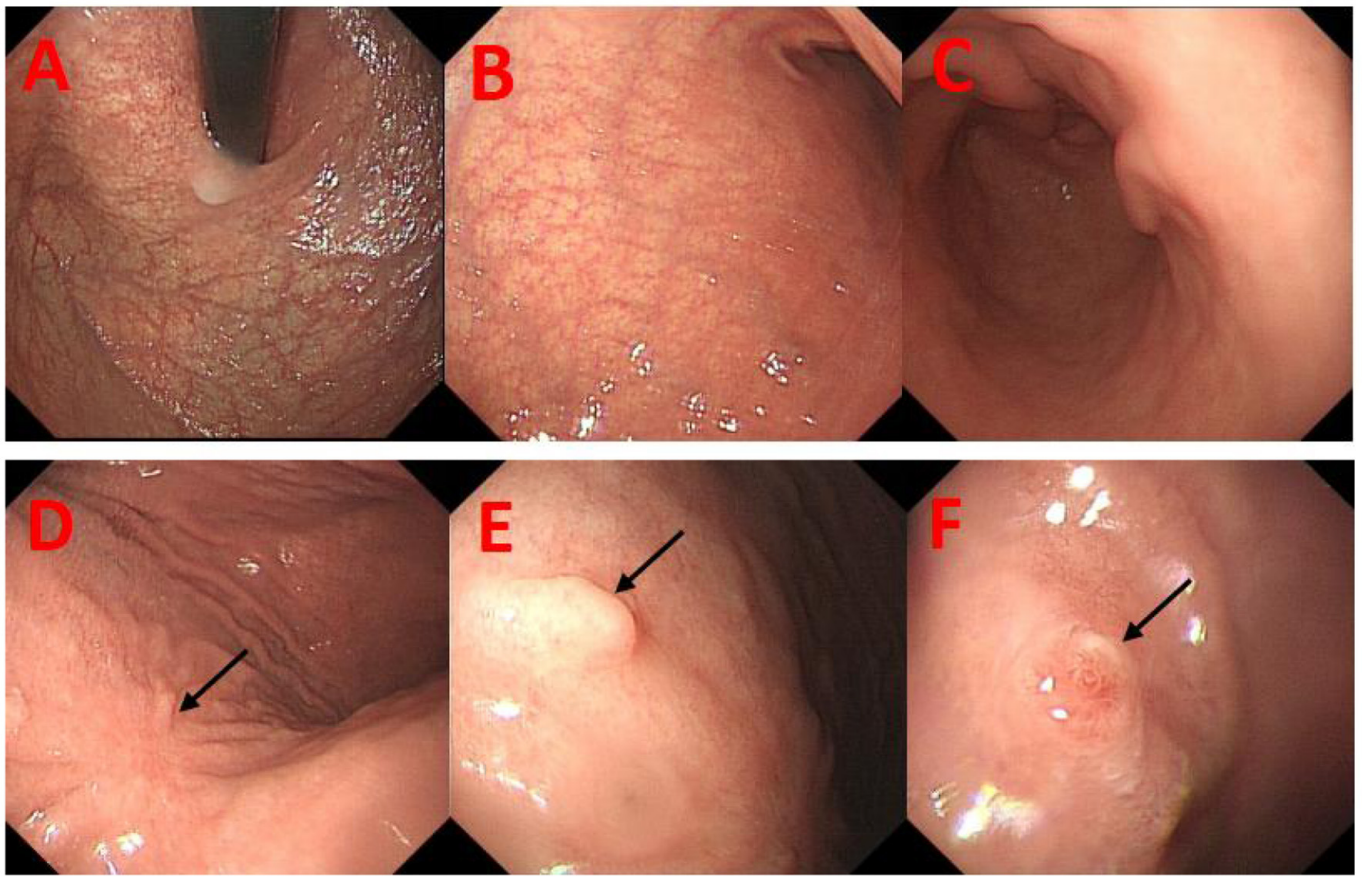
Re-examination gastroendoscopy of the patient 3 years after endoscopic submucosal dissection in March 2021. **(A, B)** There were visible submucosal vascular networks at the fundus and gastric body of the stomach. **(C)** There was no obvious atrophy of mucosa on the gastric antrum. **(D)** A surgical scar was observed in the mucosa of gastric body (black arrow). **(E, F)** In the greater curvature of the gastric body, two mucosal bulges of about 0.5 mm each were observed (black arrow).

**Figure 2:**
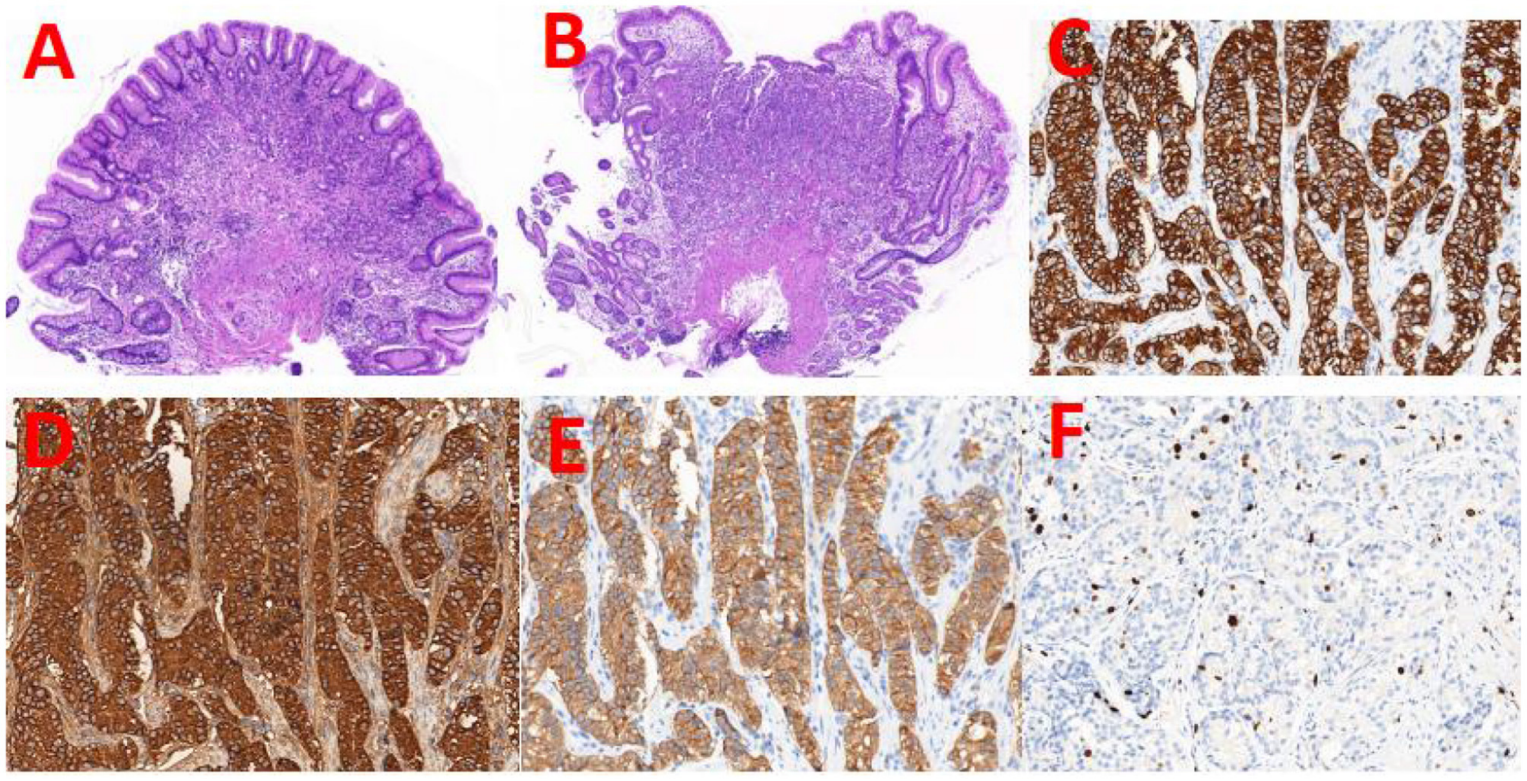
H&E staining of the two mucosal bulges and the representative images of immunohistochemical staining. **(A, B)** H&E staining of the two entire lesions (5 × magnification). The immunohistochemical staining images of the two lesions. The staining antigens were CK **(C)**, CgA **(D)**, Cyn **(E)**, and ki-67 **(F)**, respectively (40 × magnification).

Eleven days prior, the patient visited our hospital for a repeat gastroscopy. The mucosa at the gastric body and fundus was white and thin, and the submucosal vascular network was visible. Two mucosal bulges of about 0.5 × 0.5 mm in size were seen in the greater curvature of the gastric body, one of which had a red surface, with depression and erosion in the center (Figure 1). An ulcer of 0.3 cm in size was seen in the mucosa of the gastric antrum. These findings suggested a diagnosis of chronic atrophic gastritis (mainly at the gastric body and fundus), small ulcers at the gastric antrum, and mucosal bulges at the gastric body. Pathology and immunohistochemistry of the gastric body lesions indicated G2-GNET, CK(+), CgA(+), Cyn(+), and Ki67(+3%) (Figure 2). Full abdominal contrast-enhanced computed tomography did not reveal clear abnormalities in the stomach. The patient was diagnosed with G2-GNET, AIG, iron deficiency anemia, and subacute hypothyroidism.

The physician performed endoscopic submucosal dissection (ESD) on the gastric body lesions. Postoperative pathology showed chronic severe atrophic gastritis and erosion in the gastric body, with severe intestinal metaplasia. Individual atypical epithelial nests disappeared after deep incision. The patient had iron deficiency anemia and low vitamin B12. Therefore, she was given oral iron supplements, folic acid, and vitamin B12.

Postoperatively, the patient's general condition was acceptable, with no abdominal pain, melena or other discomforts. Close follow-up is currently ongoing.

## 3 Discussion

AIG, also known as type A gastritis, is an organ-specific autoimmune disease limited to the mucosa of the gastric fundus and gastric body. According to the literature, the global incidence of AIG is 0.5–4.5% (8). AIG is often overlooked and underestimated (9), as it is generally asymptomatic but may also present with dyspepsia or reflux symptoms (10, 11). In a cross-sectional study involving 379 patients with AIG, 56.7% reported gastrointestinal symptoms, the most common of which were early satiety or postprandial fullness. However, owing to its lack of specificity, AIG is rarely suspected in clinical practice based on gastrointestinal symptoms alone (12), and the patient's overall clinical manifestations are more important (13). This is also related to the endoscopist's insufficient knowledge about this disease (3). In addition, there is currently a lack of uniform standards for diagnosing AIG. Diagnosis is mainly based on serology, endoscopy, and pathology of gastric mucosal biopsy (2, 7, 14). This patient showed elevated gastrin levels and reduced PG I and PG I/II levels. Gastroscopy and biopsy pathology indicated severe mucosal atrophy and severe intestinal metaplasia of the gastric body. Immunohistochemistry of the gastric body lesions showed a Ki-67 index of 3% and epithelial markers CK (+), Syn (+), CD56 (-), and CgA (+). These findings suggested type I G2-GNET. Unfortunately, this patient was not tested for gastric autoantibodies. However, based on the factors above, the patient was diagnosed with AIG according to the Japanese diagnostic criteria (15). Type I GNET is a complication that cannot be ignored in patients with AIG, and its incidence can be as high as 15.4% (16). Under endoscopy, it usually appears as a polyp or nodule of < 1 cm in the gastric body or fundus and rarely metastasizes (1–3%) (17). In this patient, gastroscopy revealed two mucosal bulges with a diameter of about 0.5 cm, and pathology suggested GNET. However, since the lesion was relatively small, the tumor cells were thought to be essentially removed during the biopsy, and no tumor cells were found in the tissues excised during ESD.

In normal gastric physiology, the H+/K+ ATPases of parietal cells in the oxyntic gland secrete hydrochloric acid, which creates an acidic environment in the stomach. The parietal cells also produce intrinsic factor (IF), which is a protein needed for the absorption of vitamin B12 by the terminal ileum. In AIG, parietal cell antibodies (PCAs) target H+/K+ ATPases, resulting in the destruction of parietal cells by CD4+ T cells, replacement of oxyntic mucosa by pseudopyloric metaplasia or intestinal metaplasia, and decrease in oxyntic function. In addition, IF antibodies (IFAs) may also appear, thus further reducing the level of available IF. The lack of gastric acid and IF can lead to micronutrient deficiencies, including vitamin B12 and iron, and may also involve vitamin C, calcium and vitamin D deficiencies (18).

As the disease progresses, the deficiencies in vitamin B12 and iron will increase the risk of anemia and neurological disorders, such as peripheral neuropathy and subacute combined degeneration of the spinal cord (19, 20), leading to specific clinical manifestations. These symptoms can assist with the identification of AIG patients. At initial diagnosis, 37% of patients presented with hematological manifestations and 10% with neurological manifestations (21). Microcytic anemia, caused by iron malabsorption due to impaired gastric acid secretion, is currently considered an earlier and more common hematological manifestation of the disease (22). Vitamin B12 deficiency megaloblastic anemia, also known as pernicious anemia (23), may remain asymptomatic for 4–5 years, as soluble vitamin B12 (cobalamin) can be retained in the liver and used even after the onset of vitamin B12 deficiency (2). This patient had iron deficiency anemia, decreased vitamin B12, and had yet to develop hematological manifestations of pernicious anemia. Therefore, simply using the presence of pernicious anemia as a diagnostic clue may further delay the diagnosis of AIG.

AIG often coexists with other autoimmune disorders. In a study of 156 patients with AIG, about 68.6% had at least one other autoimmune disease at the time of diagnosis, and about 60.9% had autoimmune thyroiditis, followed by diabetes (19.9%) (16). Another study involving 176 patients with AIG showed that the majority of patients were female (80.7%), and 72% suffered from at least one endocrine autoimmune disease, the most common of which was autoimmune thyroid disease (35.8%) (4).

## 4 Conclusion

Clinicians who discover GNET during gastroscopy, especially in patients with concurrent iron deficiency anemia, autoimmune thyroid disease or other autoimmune diseases, should pay close attention to the presence of gastric mucosal atrophy and further perform tests for gastrin, PG, PCA, IFA, etc., to assess the possibility of AIG. This will help to improve the diagnosis rate of AIG, reduce related complications, and minimize adverse consequences rather than simply perform symptomatic treatment.

## Data Availability

The original contributions presented in the study are included in the article/supplementary material, further inquiries can be directed to the corresponding author.
